# The influence of peer group supervision during nursing education on occupational identity and well-being: Results of a mixed methods study

**DOI:** 10.3205/zma001775

**Published:** 2025-09-15

**Authors:** Stefan Wellensiek, Jan Ehlers, Michaela Stratmann

**Affiliations:** 1 Bildung & Beratung Bethel, Bielefeld, Germany; 2 Witten/Herdecke University, Faculty of Health, Witten, Germany

**Keywords:** peer group supervision, peer counselling, mixed-method, COMET, well-being, competence

## Abstract

**Background:**

The development of occupational identity is an open and dynamic process and represents an element of competence development. Measures to accompany this development include reflecting on typical professional situations, individual challenges and the professional role. Peer counselling in the format of peer group supervision appears suitable for creating the necessary opportunities for reflection.

**Aim:**

The study investigates the influence of peer group supervision on the development of occupational identity and whether peer group supervision influences psychological well-being.

**Methods:**

A group of apprentices (n=25) in the three-year nursing training programme were trained in peer group supervision and conducted it regularly over the course of a year. In a mixed-methods approach, the commitment questionnaire of the COMET instrument, the WHO-5 well-being index, progress logs and focus group interviews were used to determine the effects and influences of peer group supervision on occupational identity and well-being in the longitudinal section.

**Results:**

Peer group supervision has a moderating influence on the development of occupational identity and contributes to competence development. Psychological well-being can be improved through peer group supervision.

**Conclusions:**

Peer group supervision should be learned in a structured way and continuously be applied in vocational training.

## Zusammenfassung

**Hintergrund:**

Die Entwicklung der beruflichen Identität ist ein offener und dynamischer Prozess und stellt ein Element der Kompetenzentwicklung dar. Maßnahmen, um diese Entwicklung zu begleiten, sind die Reflexion berufstypischer Situationen, individueller Herausforderungen und der Berufsrolle. Peer-Beratung im Format der kollegialen Beratung erscheint geeignet, die notwendigen Reflexionsgelegenheiten zu schaffen.

**Fragestellung:**

Die Studie geht den Fragen nach, welchen Einfluss kollegiale Beratung auf die Entwicklung der beruflichen Identität hat und ob kollegiale Beratung das psychische Wohlbefinden beeinflusst.

**Methoden:**

Eine Gruppe von Auszubildenden (n=25) der dreijährigen Pflegeausbildung wurde in kollegialer Beratung geschult und führte diese über ein Jahr regelmäßig durch. In einem Mixed-Methods-Ansatz wurden der Commitment-Fragebogen des COMET-Instruments, der WHO-5 Wohlbefindens-Index, Verlaufsprotokolle und Fokusgruppeninterviews eingesetzt, um Effekte und Einflüsse kollegialer Beratung auf die berufliche Identität und das Wohlbefinden im Längsschnitt zu bestimmen.

**Ergebnisse:**

Kollegiale Beratung hat einen moderierenden Einfluss auf die Entwicklung beruflicher Identität und trägt zur Kompetenzentwicklung bei. Das psychische Wohlbefinden kann durch kollegiale Beratung verbessert werden.

**Schlussfolgerung:**

Kollegiale Beratung sollte strukturiert gelernt und kontinuierlich im Verlauf der Ausbildung angewendet werden.

## 1. Introduction

The development of professional identity is a dynamic and open process [1]. Its form changes in the course of professional socialisation [2]. To date, there is no standardised understanding of professional identity [3]. However, two focal points can be identified in the understanding of the term. On the one hand, it is about a personal perspective in the form of reflexive self-positioning in the context of the profession. On the other hand, there is a social perspective that emphasises belonging to a group [4]. The personal perspective tends to be translated as occupational identity, while the social perspective tends to be translated as professional identity [5]. In the context of this work, professional identity is assigned to the personal framework and is defined as assuming the professional role in order to cope with typical professional situations [6]. Therefore, the term occupational identity is used here. Characteristics of occupational identity are the degree of attachment, interest and pride in the profession as well as the desire to continue working in the profession in the future [7]. In order to develop occupational identity, it is important to analyse the professional role [6]. Reflective processes can promote and strengthen this development [8]. In this understanding, occupational identity is closely linked to competence development [6]. The development of competence and occupational identity is seen as a parallel process in which the two constructs are related to each other [9]. This relationship becomes clear in the dispositional understanding of competence, which places the elements of disposition, performance and reflection at the centre [10]. Applied to the nursing profession, nurses must reflect on dispositions and performance, e.g. knowledge of the expectations placed on them or procedures and processes in professional nursing, in order to develop an occupational identity and competence. A solidified occupational identity and thus highly developed competences have positive effects on job satisfaction and long-term retention [11].

Peer counselling offers a pragmatic and cost-effective option for reflection. The format of peer group supervision is proposed for the nursing profession [12]. This involves participants in a peer group reflecting on work-related cases in a systematic and results-orientated manner [13]. To support reflection, a key question is formulated, which is addressed using a counselling method [14]. The focus is on a self-experienced, sometimes sensitive section of one's own professional practice [15]. The aim of peer group supervision is to jointly develop a solution or approaches to professional key issues and individual challenges [16]. In contrast to medical peer review [17], peer group supervision is not an evaluation procedure for quality assurance but addresses the individual needs of those involved.

Peer group supervision provides support in overcoming professional challenges, problems or tasks [18]. New perspectives are opened up and solutions to problems are generated. Peer group supervision helps to obtain suggestions for dealing with practical professional problems and to shape the way in which training-related stress is handled [19]. In addition, peer group supervision has positive effects on the somatic well-being of nursing apprentices [20] and reduces moral stress [21]. Peer group supervision could therefore have positive effects on psychological well-being in relation to occupational stress.

As the format of peer group supervision focuses on elements of occupational identity [9] and positive effects on well-being can be assumed, the following hypothesis is used as a basis: Peer group supervision has a positive influence on the development of occupational identity and has a positive effect on psychological well-being during vocational education. 

The use of peer group supervision is legitimised by the training and examination regulations for the nursing professions. Demanding, applying and using peer counselling are defined as competences to be achieved in Annexes I and II. The framework plans of the expert commission identify peer group supervision as an element of reflection and suggest that apprentices actively and regularly participate in corresponding courses [22]. For this reason, apprentices in the nursing profession were chosen as the sample in this study.

The study examines the following key questions:

- What influence does peer group supervision have on the development of occupational identity and associated competence among apprentices in the nursing profession?
- What influence does peer group supervision have on the psychological well-being of apprentices?

## 2. Methodology

The reporting follows the guideline for “Good Reporting of A Mixed Methods Study (GRAMMS)” [23].

### 2.1. Study design

**Figure 1:**
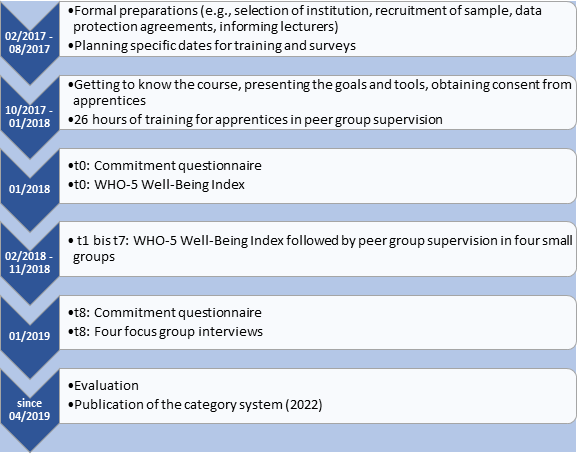
Overview of the organisational process and the use of the instruments

The study is a longitudinal study in a mixed-methods design with quantitative and qualitative instruments. The quantitative data was collected, processed and analysed in SPSS 29.0 and the qualitative data in MAXQDA 2018.2. Apprentices on a three-year nursing training course were selected as the sample. They were trained in peer group supervision and subsequently conducted seven peer group supervision sessions (see figure 1 (Fig. 1)). The specific distribution of the measurement times is shown in attachment 1 .

### 2.2. Sample 

The sample is a healthcare and nursing course in North Rhine-Westphalia in Germany with n=25 people (23 f/2 m) aged between 19 and 25. They had completed 16 months of training at the beginning of the study (t0). The final survey (t8) was at the beginning of the third year of training. The sample is relatively homogeneous: The interest and support of the parents with regard to the training is high (92% and 88% agreement) and the training corresponds to the career aspirations (84% agreement). There are differences in terms of school-leaving qualifications (eight after 10 years, seven after 12 years and nine after 13 years). Twelve people have family members in the profession, twelve do not. One person did not provide any information. Before the study began, there was no relationship between the researcher and the apprentices.

### 2.3. Data collection 

#### 2.3.1. Commitment questionnaire of the COMET instrument

The commitment questionnaire of the COMET instrument [24] was used twice (t0 and t8) to map occupational identity. The instrument contains five subscales (occupational identity, organisational identity, occupational commitment, organisational commitment, work ethics) with six items each. There are also eight questions on career choice motives (see attachment 2 ). The occupational identity subscale emphasises those cognitive and emotional dispositions that correspond to the development from novice to expert and lead to professional competence [9]. It is concretised, for example, in the items pride and interest in the profession. Threshold values (low, medium, high) are specified for categorising the mean values of the subscales [4] (see table 1 (Tab. 1)).

The questionnaire has a high internal consistency. The respective subscales achieve a Cronbach's alpha between .71 and .90 [6].

#### 2.3.2. WHO-5 well-being Index 

The WHO-5 Well-being Index [25] is used to map psychological well-being (see attachment 3 ). The instrument was used a total of eight times (t0 to t7), each time before the start of the peer group supervision. The self-assessment procedure measures a person's subjective psychological well-being regarding the last 14 days [26]. Items include, for example, the level of mood and the degree of relaxation. The rating of the items is often related to the professional experience. For example, constant arguments with superiors can influence mood or interest in work. Well-being is represented by a total value that can take on values between 0 and 25. An index value of ≤13 indicates a low level of well-being and is an indication for the specific diagnosis of depression as defined by ICD-10 [27]. The sensitivity of the scale for recognising depression has an average value of 93% and an average specificity of 64% [28]. Cronbach’s alpha is between .82 and .95 [29]. The influence of key socio-demographic factors such as age group, gender or place of residence on the reliability of the scale is low [30].

#### 2.3.3. Protocols and focus group interviews

Each counselling meeting from t1 to t7 was recorded by the participants in a standardised form (see attachment 4 ). At the end of the study (t8), three guided focus group interviews were conducted with six participants each and one with seven participants in order to uncover further influences on the development of occupational identity and individual attitudes towards peer group supervision. The composition of the interviews corresponded to the counselling groups. The interviews were transcribed and coded. A category system was available for this purpose, which is described in its genesis and is characterised by a high degree of intercoder agreement [31]. 

### 2.4. Ethical aspects 

The research project was approved by the responsible ethics committee of the University of Witten Herdecke (No. 122/2017). Informed consent with the study participants was ensured by the verbal and written provision of all relevant information and its subsequent written confirmation. 

## 3. Results

### 3.1. Occupational identity

Table 2 (Tab. 2) provides an overview of the values of all subscales at the measurement times t0 and t8 as well as a categorisation of the threshold values. 

The mean value [m] of occupational identity increases from m=13.84 (t0) to m=16.60 (t8) over the course of the study. It thus changes in its expression from low to medium. This increase is highly significant (Z=-3.309, p<.001). No other significant changes could be detected. However, the categorisation of the threshold values changed. The level of organisational identity declined from medium to low, while the others remained unchanged.

The evaluation of the focus group interviews [fg] illustrates the development of occupational identity. The apprentices grow into their professional role by naming and reflecting on specific, often stressful situations. In category 4.1.1 “specific topics and questions” [31], 69 situations were recorded. Of these, 53 were the subject of peer group supervision and were formulated as key questions. The key issues include organisational aspects (e.g. filling in at weekends), relationship aspects (e.g. dealing with conflicts in the team), skills development (e.g. problems with lack of learning success), ethical aspects (e.g. dealing with own care mistakes) and sexual harassment. Over the course of the peer group supervision, apprentices [a] become increasingly successful in describing situations, concretising key issues and expressing their thoughts on them. “And I think the more often you meet, the easier it is to talk about these problems.” (fg3, a4, 19) The apprentices rated the opportunity to talk about professional situations they had experienced positively, as these were repeated in a similar form. “I think you feel more understood in the group. Also because the others also have these problems.” (fg2, a15, 388) Solidarity with the counselling group and the other apprentices is discussed. The interviewees agree that peer group supervision should be introduced as early as possible. “I would have liked peer group supervision at the beginning, and I think it’s especially helpful when you have absolutely no idea [...] and you are faced with a lot of problems.” (fg1, a17, 292). Several participants suggest peer group supervision across training levels, in which beginners can benefit from advanced learners (fg3, a4, 278). “That would be a good way to help each other.” (fg3, a3, 24) In addition, peer group supervision should extend “[...] from the beginning of the vocational training to the end [...]” (fg3, a5, 177). These points illustrate the examination of the professional role and the associated moderating effect of peer counselling on the development of occupational identity and competence.

### 3.2. Well-being

**Figure 2:**
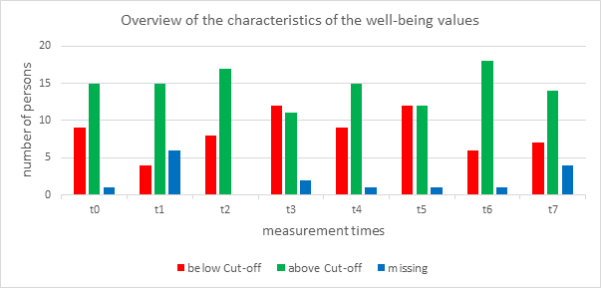
Overview of the characteristics of the well-being values (WHO-5)

The results for the WHO-5 Well-being Index are shown for each of the eight measurement times (t0 to t7) with values above (>13) and below (≤13) the cut-off score (see figure 2 (Fig. 2)).

At each time point, some people are below the cut-off value. This occurs more frequently at time points t3 and t5 (48% in each case). In the longitudinal comparison of the measurement times (Wilcoxon test), the central tendencies of the distribution for the individual persons mostly remain the same - well-being remains relatively stable. There are two exceptions: There is a significant worsening in well-being from t1 to t3 (Z=-2.236, p=.025). It should be noted that only 18 people were analysed at time t1 due to a lack of participants. In detail, there are five worsenings, no improvements and 13 constant total values between the two points in time. From t3 to t6 there is a medium significant improvement in well-being (Z=-2.121, p=.034). In this period, there was one worsening, seven improvements and 14 constant total values.

In the focus group interviews, the apprentices were confronted with the mean values of their own group’s well-being at the respective points and asked to explain the changes and influencing factors. The explanations are summarised in category 2.3.1 “explanation of well-being values” [31]. Stressful influences mentioned included general dissatisfaction in the class, a so-called mid-course low, a long school block, the time shortly before a holiday, an upcoming mid-term exam, a previous negative practical assignment, high heat in the classroom and problems with a teacher. A practical assignment that went well and the time shortly after the holiday were named as factors that relieved the pressure. The respondents were also asked whether the peer group supervision had helped them to cope with the situations mentioned. These impressions are summarised in category 4.3 “overall impression” [31]. The apprentices described the peer group supervision as a place where they could speak calmly and openly (fg1, a23, 304) and became increasingly courageous over time (fg2, a14, 156). They had noticed a positive change (fg2, a14, 158), recognised problems more objectively (fg2, a15, 404), had fun (fg4, a10, 11) and developed solutions (fg3, a1, 183). The peer group supervision was characterised by “[...] quite terrible [...]” and “[...] it takes getting used to [...]” (fg4, a9, 14) at the start to “[...] really good and helpful [...]” (fg2, a12, 356) at the end. The mutual support, respect, being taken seriously and the harmony in the group were emphasised several times. One apprentice puts it in a nutshell: “[The group] was like a little secret club that you belong to.” (fg2, a12, 256). Peer group supervision contributed to psychological well-being in these areas.

## 4. Discussion

### 4.1. Influence of peer group supervision on occupational identity 

The expression of occupational identity is not a constant variable. It changed significantly over the course of the study. The apprentices were increasingly successful in coming to terms with their professional role. The peer group supervision was able to moderate this development by the participants discussing professional situations, analysing and interpreting tasks and challenges as a peer group. Both approaches and concrete solutions were developed. The intrinsic structural characteristics of peer group supervision and the participants’ own professional situations paved the way for this. The study identifies various topics and key issues of peer group supervision. These are predominantly assigned to the domain of personal competence [10]. They reflect the specific interests and individual challenges of the apprentices and are based on intrinsic motivation in the sense of self-determination and the apprentices own experience of competence [32]. The specific topics and questions mentioned in this study are at least partially confirmed by other studies [19]. In principle, it seems desirable to promote the development of occupational identity, as it has a positive effect on the career development of individuals and leads, for example, to fewer intentions to resign [33]. In relation to the nursing profession, there is a medium correlation between long-term career retention and a positive occupational identity (r=.37; p>.001) [6]. Job satisfaction correlates with a medium effect size with the level of occuaptional identity (r=.31; p=.002) [11]. The correlation between occupational identity and overall job satisfaction is also high in other healthcare professions (r=.64; p>.001) [7]. It could therefore be relevant for individuals, institutions and society to strengthen occupational identity through peer group supervision in order to develop skills, promote job satisfaction and ensure long-term retention in the profession. The transferability of the results to other professional groups in the healthcare sector cannot be proven due to the methodological approach but seems likely. 

### 4.2. Influence of collegial counselling on well-being

During their training, apprentices experience both stressful situations (e.g. a high learning workload) and stress-relieving situations (e.g. overcoming challenges) [34]. These can have a direct effect on well-being, e.g. raising the mood or the degree of relaxation. For example, 46.3% of apprentices experience examinations as demanding and stressful [35]. Various influencing factors, e.g. a long school block, have a strong impact on well-being, e.g. a good mood. Institutions and teachers should not ignore these periodic fluctuations or dismiss them as normal, as they can influence them by organising the training. 

Based on the quantitative data, no significant changes in well-being can be demonstrated because of collegial counselling. The qualitative data indicate positive influences. Peer group supervision is a method for addressing the worsening of well-being at certain times, intercepting impending depressive moods, reducing stress and improving motivation. The reflective nature of the peer group supervision offers apprentices space to exchange ideas, experience solidarity and develop solutions. In this way, peer group supervision has a stabilising effect and contributes to individual psychological relief. The peer group is seen by the apprentices as a safe framework, like “[...] a small secret club [...]” (fg2, a12, 256) and serves as a network that catches particularly difficult situations, makes them visible and makes them workable – where other options reach their limits or fail. These aspects indicate that the well-being of the participants could be positively influenced by peer group supervision, e.g. in terms of relaxation and tension. Whether these effects extend beyond the training programme or whether strong peer groups can form in other contexts remains unclear. The transfer to other healthcare professions has not yet been clarified but is being investigated in various approaches [36].

It is striking that the individual values for well-being tend to be stable. This means that individual apprentices feel bad over a longer period and run the risk of developing depression. Peer group supervision should not be seen as the sole means of providing relief. Other appropriate measures relating to the central themes and key issues should be taken. The hypothesis that peer counselling has a positive influence on well-being can be confirmed. This is evident in the qualitative analysis, but not in the quantitative data.

### 4.3. Limitation

The sample consists of a closed course. Its composition, e.g. in terms of group size or gender distribution, could not be changed. At 25 people, the sample is small and not representative. The study relates to the second and beginning of the third year of training, which are often characterised by low motivation [37]. This could have had an impact on the participation and perception of peer counselling. The underlying concept of identity and competence, the instruments used, the training carried out and the supervision of the counselling sessions are shaped by the dispositions of the researcher. The extent to which the data is influenced by this cannot be assessed from a subjective perspective.

## 5. Conclusions

The study can prove that peer group supervision as part of nursing training has a positive influence on the development of occupational identity, competence and mental well-being. The apprentices consider implementation in the training programme to be sensible and desirable. They associate it with the development of approaches and solutions to professional challenges and experience psychological relief. Familiarity within the peer group is cited as a crucial element. Both in-course and cross-training peer group supervision sessions are offered as an option. Apprentices learn to scrutinise things, think critically, reflect on situations, understand different contexts and support each other. The contribution to the development of professional identity and competences, particularly in the personal domain, becomes clear. The implementation of peer group supervision required in the training framework [22] is thus empirically supported. Implementation in the curricula of university education also seems sensible and possible, as peer group supervision is seen as part of the task and activity profile of academically qualified nurses [38]. Research desiderata arise, for example, in the transfer to other healthcare professions at other times of training in vocational or academic programmes.

## Authors’ ORCIDs

- Stefan Wellensiek: [0009-0007-9172-9110]
- Jan Ehlers: [0000-0001-6306-4173]
- Michaela Stratmann: [0000-0002-7166-5160]

## Competing interests

The authors declare that they have no competing interests. 

## Supplementary Material

Distribution of measurement times

Commitment questionnaire

WHO-5 – Well-being Index

Minutes of the group meetings

## Figures and Tables

**Table 1:**
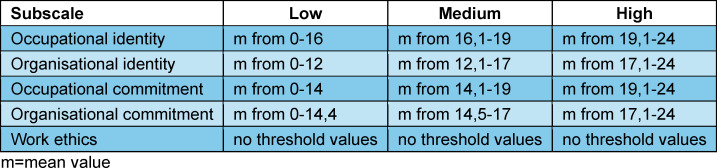
Overview of the threshold values for the mean values of the subscales [4]

**Table 2:**
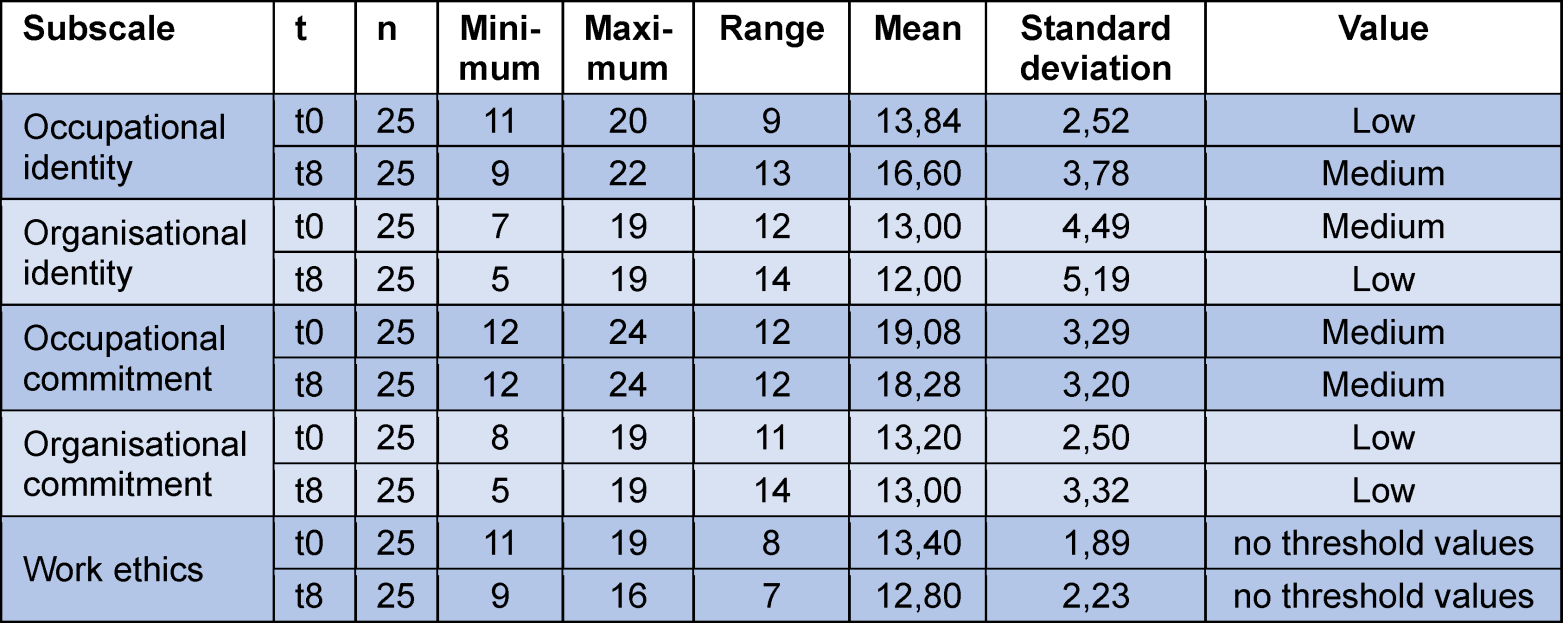
Overview of the descriptive statistics of the subscales and change in the values
