# Supplementary material for: The influence of peer group supervision during nursing education on occupational identity and well-being: Results of a mixed methods study
Source: GMS J Med Educ. 2025 Sep 15;42(4):Doc51. doi: 10.3205/zma001775 (PMC12527389; doi:10.3205/zma001775)
Supplement: Distribution of measurement times [file JME-42-51-s-001.pdf]

Attachment 1: Distribution of measurement times

Event	Date	Period of vocational training
Start of vocational training	01.10.2016	first year
Training in peer group supervision	16.10.2017-25.01.2018	second year
t0 Initial survey	25.01.2018	second year
t1 Peer group supervision	05.-12.02.2018	second year
t2 Peer group supervision	10.-11.04.2018	second year
t3 Peer group supervision	14.-15.06.2018	second year
t4 Peer group supervision	27.-28.08.2018	second year
t5 Peer group supervision	06.-07.09.2018	second year
t6 Peer group supervision	15.11-16.11.2018	third year
t7 Peer group supervision	27.11-28.11.2018	third year
t8 Focus group interview	15.-18.01.2019	third year
Completion of vocational training	30.09.2019	third year

Attachment 1 to Wellensiek S, Ehlers J, Stratmann M. *The influence of peer group supervision during nursing education on occupational identity and well-being: Results of a mixed methods study.* GMS J Med Educ. 2025;42(4):Doc51. DOI: 10.3205/zma001775
