# Supplementary material for: The influence of peer group supervision during nursing education on occupational identity and well-being: Results of a mixed methods study
Source: GMS J Med Educ. 2025 Sep 15;42(4):Doc51. doi: 10.3205/zma001775 (PMC12527389; doi:10.3205/zma001775)
Supplement: Commitment questionnaire [file JME-42-51-s-002.pdf]

Attachment 2: Commitment questionnaire

Commitment questionnaire [24]

This form of the questionnaire clarifies the allocation of the individual items to the various subscales. The version of the questionnaire used for the survey has a slightly different form and contains neither the coloured markings nor the naming of the subscales.

The statements on career choice and the recording of the highest school-leaving qualification follow on from this and serve only to complete the survey

Statement	Does not apply at all	Rather not applicable	Applies in part	Tends to apply	Completely true	
I like to tell others what profession I'm learning.	☐	☐	☐	☐	☐	Items of the occupational identity subscale
I "fit" my profession.	☐	☐	☐	☐	☐	
I would like to continue working in my profession in the future.	☐	☐	☐	☐	☐	
I am proud of my profession.	☐	☐	☐	☐	☐	
The job is like a piece of "home" for me	☐	☐	☐	☐	☐	
I'm not particularly interested in my job.	☐	☐	☐	☐	☐	
The company is like a piece of "home" for me.	☐	☐	☐	☐	☐	Items of the organisational identity subscale
I want to stay with my company in the future - even if I have the opportunity to move elsewhere.	☐	☐	☐	☐	☐	
I like to tell others about my company.	☐	☐	☐	☐	☐	
I "fit" with my company.	☐	☐	☐	☐	☐	
The future of my company is important to me.	☐	☐	☐	☐	☐	
I feel very little connection to my company.	☐	☐	☐	☐	☐	
I am interested in how my work contributes to the company as a whole.	☐	☐	☐	☐	☐	Items of the occupational commitment subscale
For me, my job means delivering quality.	☐	☐	☐	☐	☐	
I am absorbed in my work.	☐	☐	☐	☐	☐	
I know what the work I do has to do with my profession.	☐	☐	☐	☐	☐	
I sometimes think about how my work can be changed so that it can be done better or to a higher standard.	☐	☐	☐	☐	☐	
I would like to have a say in the content of my work.	☐	☐	☐	☐	☐	
I endeavour to deliver quality for my company.	☐	☐	☐	☐	☐	Ite ms

Attachment 2 to Wellensiek S, Ehlers J, Stratmann M. *The influence of peer group supervision during nursing education on occupational identity and well-being: Results of a mixed methods study.* GMS J Med Educ. 2025;42(4):Doc51. DOI: 10.3205/zma001775

Statement	Does not apply at all	Rather not applicable	Applies in part	Tends to apply	Completely true	
I want my work to contribute to the company's success.	☐	☐	☐	☐	☐	
I like to take on responsibility in the company.	☐	☐	☐	☐	☐	
Belonging to the company is more important to me than working in my profession.	☐	☐	☐	☐	☐	
I am interested in the company suggestion scheme.	☐	☐	☐	☐	☐	
The work in my company is so interesting that I often forget the time.	☐	☐	☐	☐	☐	
I am motivated, no matter what tasks I am given.	☐	☐	☐	☐	☐	**Items of the work ethics subscale**
I am reliable, no matter what tasks I am given.	☐	☐	☐	☐	☐	
I am always punctual, even when work doesn't require it.	☐	☐	☐	☐	☐	
I carry out work assignments according to instructions, even if I don't understand them.	☐	☐	☐	☐	☐	
I will nevertheless carry out instructions that I consider to be incorrect without contradiction.	☐	☐	☐	☐	☐	
For me, work means carrying out professional activities according to precise instructions.	☐	☐	☐	☐	☐	

Statement	Applies	Tends to apply	Rather not applicable	Does not apply
My parents are interested in my apprenticeship	☐	☐	☐	☐
My parents support me in my apprenticeship	☐	☐	☐	☐

Statement	Applies	Tends to apply	Rather not applicable	Does not apply
I've always wanted to do this job.	☐	☐	☐	☐
I actually wanted to learn a different profession, but I only got an apprenticeship in this profession.	☐	☐	☐	☐
If so, which one?				
Above all, I wanted to do an apprenticeship at my current company.	☐	☐	☐	☐
Friends introduced me to the profession.	☐	☐	☐	☐
Some of my family or close relatives also work in my profession.	☐	☐	☐	☐
The apprenticeship is a good basis for future studies.	☐	☐	☐	☐

My highest school-leaving qualification is a:		
	Lower secondary school leaving certificate (after 10 years)	☐
	Extended secondary school leaving certificate (after 10 years)	☐
	Intermediate school-leaving certificate (after 10 years)	☐
	Entrance qualification for studies at universities of applied sciences (after 12 years)	☐
	General qualification for university entrance (after 13 years)	☐
	Completed university degree	☐
