# Supplementary material for: The influence of peer group supervision during nursing education on occupational identity and well-being: Results of a mixed methods study
Source: GMS J Med Educ. 2025 Sep 15;42(4):Doc51. doi: 10.3205/zma001775 (PMC12527389; doi:10.3205/zma001775)
Supplement: WHO-5 – Well-being Index [file JME-42-51-s-003.pdf]

# Attachment 3: WHO-5 – Well-being Index

WHO-5 – Well-being Index [25]

This form of the questionnaire clarifies the individual items and the possible distribution of points.
The version of the questionnaire used for the survey has a slightly different format.

| **In the last two weeks…** | **All of the time** | **Most of the time** | **More than half of the time** | **Lett than half of the time** | **Some of the time** | **At no time** |
|---|---|---|---|---|---|---|
| **I have felt cheerful and in good spirits.** | 5 | 4 | 3 | 2 | 1 | 0 |
| **I have felt calm and relaxed.** | 5 | 4 | 3 | 2 | 1 | 0 |
| **I have felt active and vigorous.** | 5 | 4 | 3 | 2 | 1 | 0 |
| **I woke up feeling fresh and rested.** | 5 | 4 | 3 | 2 | 1 | 0 |
| **My daily life has been filled with things that interested me.** | 5 | 4 | 3 | 2 | 1 | 0 |

Attachment 3 to Wellensiek S, Ehlers J, Stratmann M. *The influence of peer group supervision during nursing education on occupational identity and well-being: Results of a mixed methods study.* GMS J Med Educ. 2025;42(4):Doc51. DOI: 10.3205/zma001775
