# Supplementary material for: The influence of peer group supervision during nursing education on occupational identity and well-being: Results of a mixed methods study
Source: GMS J Med Educ. 2025 Sep 15;42(4):Doc51. doi: 10.3205/zma001775 (PMC12527389; doi:10.3205/zma001775)
Supplement: Minutes of the group meetings [file JME-42-51-s-004.pdf]

Attachment 4: Minutes of the group meetings

Minutes of the group meetings of Group G __

Please complete these minutes at the end of the meeting and place them in the drop box together with the other questionnaires.

Date of the meeting: ___________________

Start of the meeting (exact time): ___________________

End of meeting (exact time): ___________________

Number of situations **discussed**: ___________________

Number of situations **worked** on: ___________________

Situation I

Please tick the box that best describes how the roles were distributed!

**Pseudonymous**	**Roles**			
**BA06JO**	☐ Storyteller	☐ Moderator	☐ Consultant	☐ Secretary
**EM05FA**	☐ Storyteller	☐ Moderator	☐ Consultant	☐ Secretary
**MA20FR**	☐ Storyteller	☐ Moderator	☐ Consultant	☐ Secretary
**MA26BE**	☐ Storyteller	☐ Moderator	☐ Consultant	☐ Secretary
**NA16FA**	☐ Storyteller	☐ Moderator	☐ Consultant	☐ Secretary
**NA30NI**	☐ Storyteller	☐ Moderator	☐ Consultant	☐ Secretary
**SU28RA**	☐ Storyteller	☐ Moderator	☐ Consultant	☐ Secretary

What was the topic of the peer group supervision??

____

Which supervision method did you use?

____

Please write down the key question here!

____

____

Attachment 4 to Wellensiek S, Ehlers J, Stratmann M. *The influence of peer group supervision during nursing education on occupational identity and well-being: Results of a mixed methods study.* GMS J Med Educ. 2025;42(4):Doc51. DOI: 10.3205/zma001775
